# AI Evaluation of Imaging Factors in the Evolution of Stage-Treated Metastases Using Gamma Knife

**DOI:** 10.3390/diagnostics13172853

**Published:** 2023-09-04

**Authors:** Calin G. Buzea, Razvan Buga, Maria-Alexandra Paun, Madalina Albu, Dragos T. Iancu, Bogdan Dobrovat, Maricel Agop, Viorel-Puiu Paun, Lucian Eva

**Affiliations:** 1 Clinical Emergency Hospital “Prof. Dr. Nicolae Oblu”, 700309 Iasi, Romania; calinb2003@yahoo.com (C.G.B.); bugarazvan@yahoo.com (R.B.); madalina.alb@gmail.com (M.A.); dt_iancu@yahoo.com (D.T.I.); bogdan.dobrovat@yahoo.com (B.D.); elucian73@yahoo.com (L.E.); 2 National Institute of Research and Development for Technical Physics, IFT, 700050 Iasi, Romania; 3 Department of Medical Oncology and Radiotherapy, University of Medicine and Pharmacy “Grigore T. Popa”, 700115 Iasi, Romania; 4 Division Radio Monitoring and Equipment, Section Market Access and Conformity, Federal Office of Communications OFCOM, Avenue de l’Avenir 44, CH-2501 Biel/Bienne, Switzerland; maria_paun2003@yahoo.com; 5 Regional Institute of Oncology, 700483 Iasi, Romania; 6 Physics Department, Technical University “Gheorghe Asachi” Iasi, 700050 Iasi, Romania; m.agop@yahoo.com; 7 Physics Department, Faculty of Applied Sciences, University Politehnica of Bucharest, 060042 Bucharest, Romania; 8 Romanian Scientists Academy, 54 Splaiul Independentei, 050094 Bucharest, Romania; 9 Faculty of Dental Medicine, Universitatea Apollonia, 700399 Iasi, Romania

**Keywords:** stereotactic radiosurgery, radiomics, brain metastasis, treatment response, machine learning

## Abstract

Background: The study investigated whether three deep-learning models, namely, the CNN_model (trained from scratch), the TL_model (transfer learning), and the FT_model (fine-tuning), could predict the early response of brain metastases (BM) to radiosurgery using a minimal pre-processing of the MRI images. The dataset consisted of 19 BM patients who underwent stereotactic-radiosurgery (SRS) within 3 months. The images used included axial fluid-attenuated inversion recovery (FLAIR) sequences and high-resolution contrast-enhanced T1-weighted (CE T1w) sequences from the tumor center. The patients were classified as responders (complete or partial response) or non-responders (stable or progressive disease). Methods: A total of 2320 images from the regression class and 874 from the progression class were randomly assigned to training, testing, and validation groups. The DL models were trained using the training-group images and labels, and the validation dataset was used to select the best model for classifying the evaluation images as showing regression or progression. Results: Among the 19 patients, 15 were classified as “responders” and 4 as “non-responders”. The CNN_model achieved good performance for both classes, showing high precision, recall, and F1-scores. The overall accuracy was 0.98, with an AUC of 0.989. The TL_model performed well in identifying the “progression” class, but could benefit from improved precision, while the “regression” class exhibited high precision, but lower recall. The overall accuracy of the TL_model was 0.92, and the AUC was 0.936. The FT_model showed high recall for “progression”, but low precision, and for the “regression” class, it exhibited a high precision, but lower recall. The overall accuracy for the FT_model was 0.83, with an AUC of 0.885. Conclusions: Among the three models analyzed, the CNN_model, trained from scratch, provided the most accurate predictions of SRS responses for unlearned BM images. This suggests that CNN models could potentially predict SRS prognoses from small datasets. However, further analysis is needed, especially in cases where class imbalances exist.

## 1. Introduction

Gamma Knife stereotactic radiosurgery (GKSRS) is a non-invasive technique used to treat brain tumors, vascular malformations, and other neurological conditions. Its history dates back to the 1940s when neurosurgeon Lars Leksell developed the concept of radiosurgery. In the 1950s, Leksell and Borje Larsson created the first Gamma Knife prototype at the Karolinska Institute in Sweden. The machine uses 192–201 cobalt-60 sources to deliver precise brain radiation [1].

The first clinical use of Gamma Knife treatment was in 1968, and it began gaining popularity in the 1970s and 1980s [2]. Initially, it treated hard-to-reach brain tumors and vascular malformations. Now, it addresses neurological conditions like trigeminal neuralgia, epilepsy, and Parkinson’s disease [3].

Over time, the Gamma Knife has seen technological improvements, including computerized treatment planning and image guidance. Today, it is used in over 700 medical centers worldwide, treating over one million patients per year [1,2,3,4].

Machine learning (ML) is a subset of AI, enabling computer systems to learn from experience without explicit programming. ML uses algorithms trained on data to recognize patterns and make decisions as humans do [5,6].

The three main ML algorithms are:

1. Supervised learning: Maps input data to known output data for predictions, e.g., linear regression, decision trees, and neural networks.
2. Unsupervised learning: Identifies patterns and relationships in data, without supervision, e.g., clustering and principal component analysis.
3. Reinforcement learning: Learns through trial and error, optimizing for cumulative rewards, e.g., Q-learning and deep reinforcement learning.

ML finds applications in natural language processing, image recognition, predictive maintenance, fraud detection, and recommendation systems [5].

Deep learning, a subfield of ML, employs multi-layer neural networks for complex tasks like image and speech recognition. It excels in computer vision, speech recognition, and natural language processing [7,8].

There are various types of deep learning algorithms for different problems [7,8]:

1. Convolutional neural networks (CNNs): Used for image and video recognition, and for analyzing local patterns.
2. Recurrent neural networks (RNNs): For sequential data, such as speech recognition and time series analysis.
3. Generative adversarial networks (GANs): Generate data similar to a given dataset, such as realistic images.
4. Autoencoders: Tasked with image/text compression, feature extraction, and denoising.

Many other algorithm variations exist to solve diverse problems [7,8]. Transfer learning (TL) is a technique in which a pre-trained model kickstarts a new task. The model first learns general features from a large dataset, then trains on a smaller, specific sample. TL is beneficial with limited labeled data, leveraging the pre-trained model’s knowledge [9,10]. It is used in computer vision, natural language processing, and speech recognition [11,12].

There are some new methods, like RCNN (region-based convolutional neural network) [13] and attention models [14], which are two different techniques commonly used in computer vision tasks. While they might not be the most typical choices for addressing the problem of early response prediction of brain metastases, they could potentially be applied in creative ways to enhance the performance of predictive models for this specific medical imaging task. RCNN is a family of methods used for object detection and localization in images. It involves dividing an image into multiple regions (proposals) and processing each region separately to detect and classify objects within them. In the context of brain metastases prediction, RCNN-like approaches could be adapted to identify and classify different regions of interest (ROIs) within medical images that indicate the presence of metastatic growth. These regions could correspond to areas with abnormal features indicative of metastases. By processing these ROIs separately, the model could potentially learn to detect early signs of metastases that might be overlooked by more traditional image analysis methods. Attention mechanisms have gained popularity in various deep learning applications, including computer vision and natural language processing. Attention mechanisms help models focus on the most relevant parts of the input data when making predictions. In the context of brain metastases prediction, attention mechanisms could be used to guide the model’s focus to specific regions within the medical images that are more likely to contain early signs of metastases. This could be particularly helpful in identifying subtle patterns or anomalies that might not be immediately apparent to human observers or traditional image analysis methods.

It is important to note that neither RCNN nor attention models are directly designed for the early response prediction of brain metastases. The typical approach for medical image analysis involves techniques such as convolutional neural networks (CNNs) and other deep learning architectures specifically tailored for image classification and segmentation tasks. However, the application of RCNN-like techniques and attention mechanisms in this context could be explored as part of a more advanced and innovative approach to improve the sensitivity and accuracy of early response prediction for brain metastases.

The success of these techniques would depend on factors such as the availability of labeled data, the complexity of the metastases detection task, and the computational resources available for model training and evaluation. It is recommended to work closely with domain experts in radiology and medical imaging when designing and evaluating such models to ensure their clinical relevance and efficacy.

GKSRS is a non-invasive radiation therapy for brain conditions. Machine learning and deep learning enhance SRS in multiple ways [15,16,17,18,19]:

1. Treatment planning: ML helps identify brain structures in medical images (MRI) for precise target delineation.
2. Dose optimization: ML optimizes radiation doses during planning, balancing efficacy and tissue protection.
3. Prediction of outcomes: ML predicts SRS treatment outcomes based on patient characteristics.
4. Quality assurance: ML automates error detection in treatment delivery, enhancing safety and efficacy.
5. Treatment evaluation: ML assesses treatment effectiveness through patient data, refining protocols.

In summary, machine learning and deep learning improve Gamma Knife stereotactic radiosurgery, leading to a more efficient healthcare system [18,19].

In this work, we utilize Google Colaboratory (Google Colab), a cloud-based platform for Python code development via Jupyter notebooks. It offers a free environment for researchers, data scientists, and ML practitioners to analyze data, perform machine learning tasks, etc. [20]. Google Colab boasts features such as access to free GPUs and TPUs for model training, integration with Google Drive for storage and notebook sharing, and real-time code cell execution with instant feedback. Popular Python libraries like TensorFlow, Keras, and PyTorch are supported [18,19].

Data augmentation is employed in ML and computer vision to expand training datasets with varied samples, addressing limited data and overfitting [21,22,23,24]. ML techniques include flipping, rotating, scaling, cropping, adding noise, and adjusting brightness/contrast. These transformations enhance model robustness and accuracy, with care taken to maintain data representativeness [24].

Unbalanced datasets pose challenges in ML when one class vastly outweighs the others. Approaches to address this issue include resampling (oversampling/undersampling), class weighting, cost-sensitive learning, ensemble methods, and data augmentation [25,26,27,28,29]. The appropriate method depends on the problem and dataset, necessitating evaluation of all classes for accurate results [25,26,27,28,29].

Deep learning employs early stopping and callback lists to improve performance and prevent overfitting. Early stopping halts training when validation performance degrades after a set number of epochs, guarding against overfitting. Callback lists execute functions during training, enabling customization and the implementation of techniques like model checkpointing and learning rate scheduling [25,26,27,28,29]. Keras supports both early stopping and callback lists, enhancing model training and performance.

**Figure 1:**
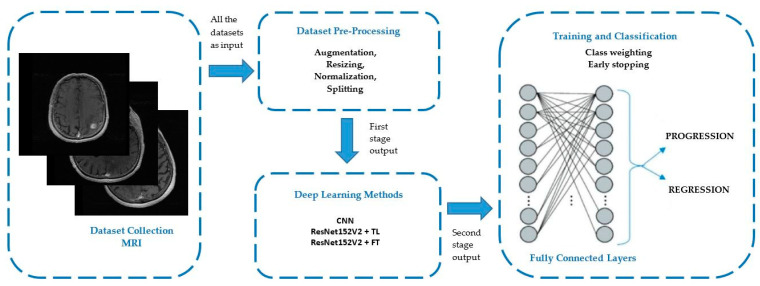
Diagram of AI workflow of prognostic factors in the evaluation of stage-treated metastasis based on medical imaging with the Gamma Knife treatment machine from our department.

In the following sections, we present an AI evaluation of prognostic factors in the evolution of stage-treated metastases based on medical imaging with the Gamma Knife treatment machine from our department, as depicted in the diagram from Figure 1.

Our present goals are as follows:

1. Gather and preprocess data: Collect MRI scans from patients with stage-treated metastases who underwent Gamma Knife treatment. Preprocess the data to ensure its analysis readiness, addressing data imbalance using augmentation techniques like SMOTE.
2. Identify prognostic factors: Use domain expertise and existing research to identify potential factors influencing metastases evolution, including tumor size, location, shape, patient demographics, and clinical history.
3. Develop the AI model: Select a suitable deep learning algorithm and train it on preprocessed data to predict metastases evolution likelihood, considering the identified prognostic factors and Gamma Knife treatment specifics. Three methods are employed: CNN model from scratch, CNN model with transfer learning, and CNN model with fine-tuning.
4. Evaluate model performance: Test the AI model on separate data to assess its predictive capabilities using metrics like accuracy, sensitivity, specificity, confusion matrix, and receiver operating characteristics.
5. Interpret results: Analyze AI model outputs to identify the most important prognostic factors affecting metastases evolution in Gamma Knife-treated patients, using visualizations and statistical analysis to explore relationships between factors.
6. Validate findings: Verify AI model results with additional data and compare predictions against outcomes of real Gamma Knife-treated patients.
7. Communicate results: Present the AI evaluation findings clearly, highlighting crucial prognostic factors and their implications for metastases treatment with the Gamma Knife.

***Research Gaps and Critical Limitations***:

This paper’s study regarding the use of deep learning techniques to predict metastases evolution post-treatment represents a significant step forward; however, there are notable research gaps and limitations that should be acknowledged:

***Sample Size and Generalization***: One critical limitation is the relatively small sample size used in the study. This raises concerns about the model’s ability to generalize to a larger patient population and diverse clinical settings. Addressing this gap is crucial to ensure the model’s robustness and reliability.

***Single-Center Design and Bias***: The single-center design of the study introduces the potential for institutional biases and limitations in regards to the diversity of patient cases. Multicenter studies or more diverse datasets are needed to validate the model’s effectiveness across different healthcare settings.

***Retrospective Nature and Confounding Factors***: The retrospective nature of the data collection may introduce confounding factors that could impact the accuracy and generalizability of the deep learning model’s predictions. Future research should consider prospective designs to minimize such biases.

***Motivation, Contribution, and Novelties***:

The motivation of the paper lies in leveraging deep learning techniques for predicting treatment outcomes in patients with stage-treated metastases who underwent Gamma Knife radiosurgery. The paper’s contributions and novelties include:

***Clinical Application of Deep Learning***: The paper introduces the novel application of deep learning in predicting metastases evolution, bridging the gap between advanced machine learning techniques and clinical decision making.

***Accurate Outcome Prediction***: The study demonstrates that deep learning algorithms can accurately predict treatment outcomes post-Gamma Knife radiosurgery. This insight has significant clinical implications for enhancing treatment decision-making processes.

***Future Research***:

The identified limitations and the potential impact of the paper’s findings suggest several avenues for future research:

***Validation in Larger Cohorts***: Future studies should aim to validate deep learning models using larger and more diverse patient cohorts. This will enhance the model’s reliability and ability to generalize to a broader patient population.

***Multi-Center Studies***: Conducting multicenter studies across different healthcare institutions can help mitigate biases associated with a single-center design and improve the model’s robustness in various clinical settings.

***Prospective Studies***: Prospective studies can offer more controlled data collection and minimize retrospective biases, thereby strengthening the validity of the deep learning model’s predictions.

***Model Interpretability***: Exploring techniques for explaining the deep learning model’s predictions could enhance its clinical utility by providing insights into the factors driving specific outcomes.

***Generalizability to Other Clinical Contexts***: Given the success of the model in predicting metastases evolution, investigating the adaptability of deep learning algorithms to other clinical contexts could expand the scope of its application.

***Ethical Considerations***: Future research should delve into the ethical implications of using AI-driven predictions in medical decision making, ensuring that patient autonomy, consent, and privacy are upheld.

***Integration into Clinical Workflow***: As mentioned in the context of an upcoming paper, developing an application for deploying the model in a clinical setting would require research into user interface design, real-time processing, and seamless integration with existing healthcare systems.

In summary, while the paper contributes valuable insights into using deep learning for predicting treatment outcomes in metastases patients, addressing its limitations and pursuing further research avenues will enhance the reliability, generalizability, and practicality of the proposed approach.

## 2. Materials and Methods

### 2.1. Ethics

All experiments were carried out in accordance with relevant guidelines and regulations. The study used only pre-existing medical data; therefore, patient consent was not required, and since it was retrospective, there was no need of approval from the Ethics Committee of Clinical Emergency Hospital “Prof. Dr. Nicolae Oblu” Iasi. 

### 2.2. Patients

From July 2022 to February 2023, in the Stereotactic Radiosurgery Laboratory, Prof. Dr. N. Oblu Emergency Clinical Hospital, Iasi, 19 patients with single metastases were staged treated according to a treatment scheme of 30 Gy administered in 3 sessions (S1, S2, S3) of 10 Gy at 2-week intervals. Among the 19 patients, 5 were female and 14 male, aged between 43 and 80 years. All treated patients had a Karnofsky score of at least 70, and the initial tumor volumes before the first treatment session varied from 2 to 81 cm^3^, with an average of 16 cm^3^. The primary site of the 19 metastases treated was bronchopulmonary neoplasm in 14 cases, breast neoplasm in 3 cases, laryngeal neoplasm and prostate neoplasm, in one case each. 

After the treatment, only one patient showed a clear regression of the lesion using the three-session treatment scheme, with another three being observed as a fluctuating progression and regression lesion case. 

### 2.3. MRI Data Acquisition

All MRI examinations were performed on a 1.5 Tesla whole-body scanner (GE SIGMA EXPLORER) that was equipped with the standard 16-channel head coil. The MRI study protocol consisted of:

1. The conventional anatomical MRI (cMRI) protocol for clinical routine diagnosis of brain tumors included, among others, an axial fluid-attenuated inversion recovery (FLAIR) sequence, as well as a high-resolution contrast-enhanced T1-weighted (CE T1w) sequence.
2. The advanced MRI (advMRI) protocol for clinical routine diagnosis of brain tumors was extended by axial diffusion-weighted imaging (DWI; b values 0 and 1000 s/mm^2^) sequence and a gradient echo dynamic susceptibility contrast (GE-DSC) perfusion MRI sequence, which was performed using 60 dynamic measurements during administration of 0.1 mmol/kg-bodyweight gadoterate meglumine.

### 2.4. Workflow

#### 2.4.1. Basic Imports

In Python, we imported fundamental libraries for scientific computing, data manipulation (NumPy and Pandas), machine learning (TensorFlow), and data visualization (Seaborn and Matplotlib).

#### 2.4.2. Image Data Processing

The BrainMet Image Dataset, consisting of three subfolders: TRAIN, TEST, and VAL, is stored in the cloud on Google Drive. For training, the train_path is used; for testing, the test_path is used; and for validation, the valid_path is used. The train subfolder contains 2865 MRI brain metastasis images, with 2083 images of regression class ‘1’ and 782 images of progression class ‘0’. The test subfolder contains 874 images, with 230 images of regression class ‘1’ and 85 images of progression class ‘0’. The val subfolder contains 14 images, with 7 images of regression class ‘1’ and 7 images of progression class ‘0’.

##### Techniques to Overcome Insufficient Data from our Image Database

As discussed in Section 1, Introduction, data augmentation is a technique to artificially increase the size of a dataset by applying image augmentation methods to the existing training data. Its use is crucial when dealing with small imaging databases, as it improves the model’s training ability by subjecting the data to various image processing techniques. This increases the model’s accuracy and enhances its capability to predict cases. In the medical image recognition field, data augmentation plays a vital role by applying small transformations to existing data. This approach is especially important to address privacy regulations that may limit the sharing of medical data.

Transfer learning is another alternative approach that utilizes pre-trained state-of-the-art CNN models, like those trained on the ImageNet dataset. It is known to achieve higher performance compared to training CNNs from scratch (full-training) [30,31].

In the following material, you will find Table 1, with the splitting of the BrainMet image dataset in TRAIN, TEST, VAL sets, and the number of images belonging to the two classes: PROGRESSION—class 0, and REGRESSION—class 1.

##### Addressing the Unbalanced Dataset Issue Which Is Present in This Study

Class weights are typically used in the calculation of the loss function. The loss function is a measure of the difference between the predicted values and the actual values, and is used to update the model’s parameters during training. By assigning higher weights to the minority class, the loss function places more emphasis on correctly classifying these samples. The class weights can be manually specified or automatically computed, based on the class frequencies in the training data.

Our image dataset is highly unbalanced, with 2320 images from the regression class ‘1’ and only 874 from the progression class ‘0.’ To address this issue, we use class weighting (see Section 1, Introduction, for details).

Class weighting is a technique in deep learning that adjusts the contribution of different classes in the loss function during training. In classification tasks with imbalanced classes, the model may become biased towards the majority class, leading to poor performance regarding the minority class. To overcome this, class weights are assigned to give more importance to the minority class during training. In our study, the regression class receives less weight (0.69) compared to the progression class (1.83), as illustrated in the following output: 

{0: 1.83, 1: 0.69}

Class weights are typically used in the loss function calculation, which measures the difference between predicted and actual values and guides the model’s parameter updates during training. By giving higher weights to the minority class, the loss function focuses more on correctly classifying these samples. Class weights can be manually specified or automatically computed, based on the class frequencies in the training data.

#### 2.4.3. Model-1: Convolutional Neural Network Model from Scratch (CNN_Model)

A convolutional neural network (CNN) is a variant of multi-layer perceptrons (MLPs) designed for 2-D imaging tasks. It comprises three layers: convolutional, subsampling, and output. The convolutional layer passes results to the next layer through convolution, function expression, and feature maps. Subsampling layers follow convolutional layers, reducing feature map size while preserving information between features.

We will briefly discuss the use of the CNN approach in addressing the problem of early response prediction of brain metastases.

** Feature Learning from Images: ** CNNs are particularly effective in image analysis tasks due to their ability to automatically learn hierarchical features from raw pixel values. In the context of brain metastases prediction, CNNs can learn to identify patterns, textures, and shapes that are indicative of early metastatic growth. This feature learning process reduces the need for manual feature engineering, which can be complex and time-consuming.

** End-to-End Learning: ** CNNs enable end-to-end learning, meaning that the model learns a mapping directly from input images in order to make predictions. This is advantageous because it optimizes the entire pipeline simultaneously, without requiring intermediate steps. For the early response prediction of brain metastases, this end-to-end approach allows the model to learn complex relationships between image features and the likelihood of progression or stagnation.

** Transfer Learning: ** Transfer learning involves using pre-trained models on large datasets and fine-tuning them for specific tasks. The use of a modified ResNet152V2 architecture, previously trained on colored images, illustrates this advantage. Leveraging a pre-trained model speeds up the convergence process and allows the model to capture general image features that could be relevant to brain metastases prediction.

** Availability of Labeled Data: ** CNNs can perform well with a moderate amount of labeled data. In medical imaging, acquiring large datasets with accurate labels can be challenging due to the need for expert annotations. CNNs can still yield meaningful results, even with relatively smaller datasets, making them suitable for medical applications in which data availability might be limited.

** Robustness to Variability: ** CNNs are designed to handle various levels of variability in images, including changes in lighting, orientation, and scale. In the case of brain metastases, images can exhibit variations in terms of image quality, patient positioning, etc. The hierarchical features learned by CNNs allow them to capture relevant information, despite these variations.

** Interpretability and Visualization: ** CNNs can provide insights into their decision-making process through techniques like feature visualization and heatmaps. This interpretability can be crucial in medical applications in which understanding why a model makes a particular prediction is important for gaining trust and clinical acceptance.

Accordingly, the main model in this project is a CNN network designed from scratch. The network uses convolution, max pooling, and dense layers, and it is trained based on input weights (images).

The process starts with a lower filter size and gradually increases layer-wise. The kernel/filter size is [3X3], and ReLU is used as the activation function. The input shape represents the dimensions of the MRI image (height, width, and color channel). Although MRI images are in grayscale, we assume a color channel of “3” because the network used (ResNet152V2) was previously trained on colored images. One could also choose 1 as the color channel for grayscale images.

After each convolution layer, a [2X2] max pooling layer is added to decrease data size and processing time. The network consists of three blocks: Block-1, with a 1X16 Conv2D layer and one MaxPooling2D layer; Block-2, with 2X32 Conv2D layers and two MaxPooling2D layers after each convolution layer; and Block-3, with 2X64 Conv2D layers and two MaxPooling2D layers after each convolution layer.

The final layer is a flatten layer, followed by dense layers for classification/prediction. The sigmoid function is used in the last layer, since the problem is a binary classification (stagnation or progression),with one output unit.

To compile the model, three main parameters are required: (1) learning rate (optimizer), (2) loss function (binary_crossentropy), and (3) metrics (accuracy) to evaluate the training and validation sets’ loss and accuracy. The Adam optimizer is mainly used in this case, as it provides adaptive learning rates for different parameters.

The model architecture we use is a convolutional neural network (CNN) based on the ResNet152V2 architecture, which we are adapting for the task of early response prediction of brain metastases using MRI images. Our model selection, loss function, training strategy, and other relevant aspects, are broken down as follows:

** Model Architecture: ** We are using a modified ResNet152V2 architecture, which is a deep CNN architecture known for its strong performance for various computer vision tasks. By customizing the architecture to our specific problem, we are leveraging the hierarchical feature extraction capabilities of the CNN layers. The gradual increase in filter size and the introduction of max pooling layers help to capture increasingly complex features from the MRI images.

** Loss Function: ** Binary cross-entropy loss (binary_crossentropy) is a suitable choice for binary classification tasks like ours (regression or progression). It quantifies the difference between predicted probabilities and true labels, encouraging the model to produce higher probabilities for the correct class and lower probabilities for the incorrect class. This loss function is widely used in binary classification scenarios.

** Training Strategy: ** Our training strategy involves using the Adam optimizer, with a specified learning rate. Adam is an adaptive optimization algorithm that adjusts the learning rates for each parameter based on the historical gradients. This can help the model converge faster and find a good set of weights. Additionally, we are using accuracy as a metric to evaluate the model’s performance on both training and validation sets. It is important to monitor not only the training accuracy, but also the validation accuracy to detect overfitting, as we did.

** Model Layers and Activation Function: ** The use of ReLU activation functions after each convolutional layer is a common practice. ReLU helps introduce non-linearity into the model and can improve the network’s ability to capture complex relationships in the data. Max pooling layers after each convolutional layer reduce the spatial dimensions of the feature maps, helping to decrease the computational load and retain essential features.

** Flatten and Dense Layers: ** The final layers include a flatten layer, followed by dense layers for classification. This is a typical setup in which the spatial information is flattened and then passed through fully connected layers for classification. Using a sigmoid activation function in the last layer is appropriate for binary classification, as it produces an output in the range of [0, 1], representing the probability of the positive class.

Overall, our model architecture, loss function, training strategy, and other design choices seem reasonable for the task of early response prediction of brain metastases using MRI images.

The word “parameters” refers to the count of weights learned during our training process. These parameters play a crucial role in the model’s predictive power, as they are updated layer-wise using the back propagation method, driven by the optimization technique, which is Adam learning, in this case. As seen in Table 2, we have three columns: (1) Layer (type), (2) Output Shape, and (3) Param #, which represents the parameters. For each layer, the parameters are calculated and generated. The input layer, which is just an assigned input image shape, is not listed in the table and does not have learnable parameters.

##### Fitting and Evaluating the Model (CNN_Model)

**Figure 2:**
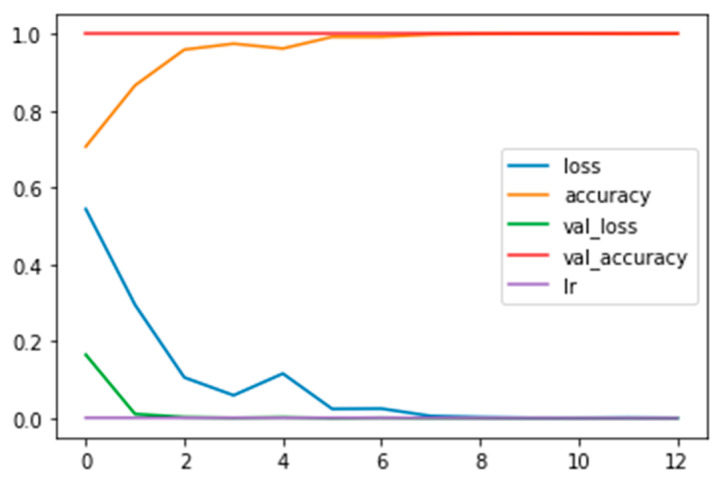
Plot of the training and validation and the loss and accuracy curves, along with the learning rate (see the legend) for the CNN_model from scratch.

In Figure 2, the plot of the training and validation and the loss and accuracy curves, along with the learning rate (see the legend) for the CNN_model from scratch, is displayed.

The accuracy of the CNN_model:

The testing accuracy is: 98.41269850730896%.

**Figure 3:**
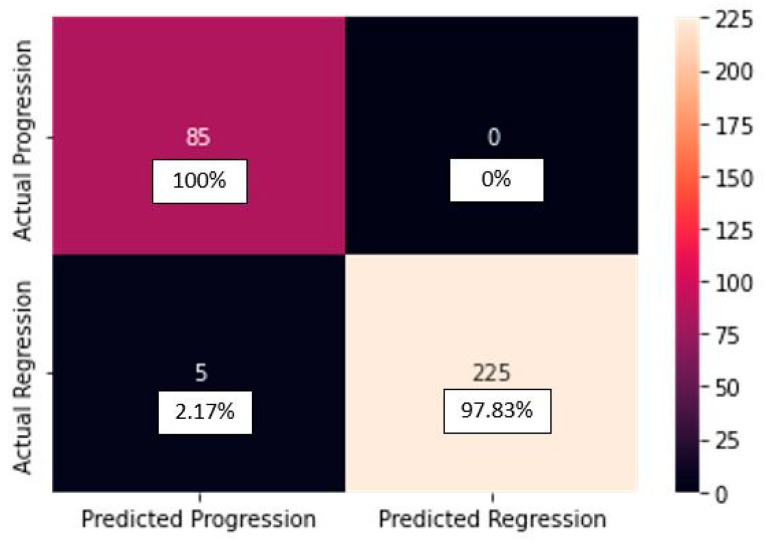
Confusion matrix for the CNN_model from scratch.

The confusion matrix of the CNN_model:

In Figure 3, the confusion matrix for the CNN_model from scratch can be seen.

From the confusion matrix for the CNN_model from scratch, we can see that from the 85 progression cases, the model predicted 85 cases correctly. From the 230 regression cases, the model predicted 225 cases correctly, making only 5 mistakes, predicting progression for cases actually belonging to the regression class (false negatives). The classification report for the CNN_model from scratch is depicted in Table 3.

**Figure 4:**
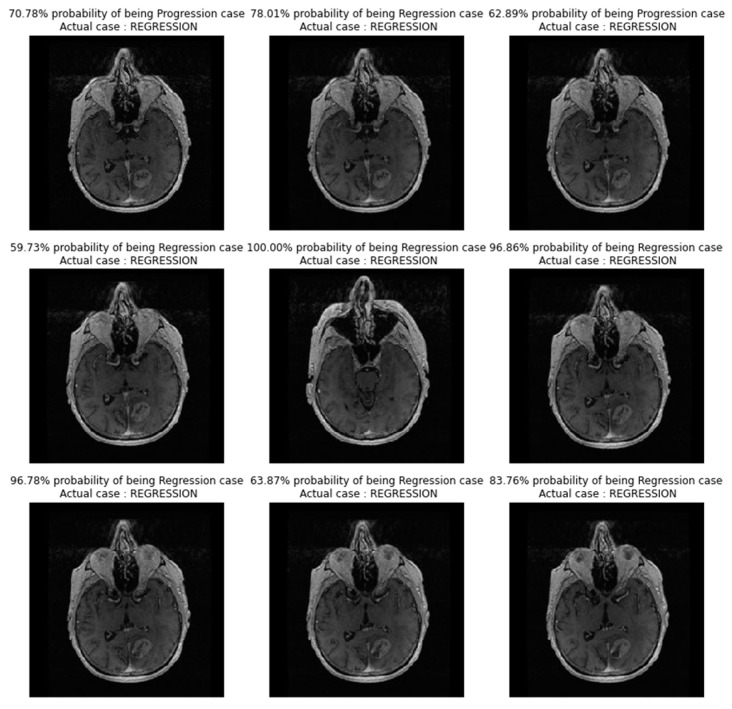
Images showing actual cases versus the predicted cases, with probability of prediction, for the CNN_model from scratch on unseen images.

In Figure 4, images showing actual cases versus the predicted cases, with the probability of prediction, for the CNN_model from scratch on unseen images, are presented.

#### 2.4.4. Model-2: Transfer Learning (TL_Model)

In our study, we employed transfer learning (TL) using a pretrained model (see Table 4), specifically ResNet152V2, which was pretrained on the ImageNet dataset. 

Residual network (ResNet) networks are deep networks that avoid vanishing gradient issues through “skip connection”. ResNet has different models with varying numbers of layers, such as ResNet50, ResNet50V2, ResNet101, ResNet101V2, ResNet152, and ResNet152V2.

In ResNet, convolution layers and other methods are used, but the key is the “skip connection” that adds the original input to the output of the convolution block. This skips some layers, preventing the gradient from vanishing. 

Weights download for the ResNet model:

234545216/234545216 [==============================]—2 s 0 us/step

As the featured learning layers are frozen, the parameters of these layers are also predetermined. Finally, the classified layer parameters are obtained by the retrained layer. All the parameter rules and formulas are similar to those mentioned in Model-1.

##### Fitting and Evaluating the Model (TL_Model)

**Figure 5:**
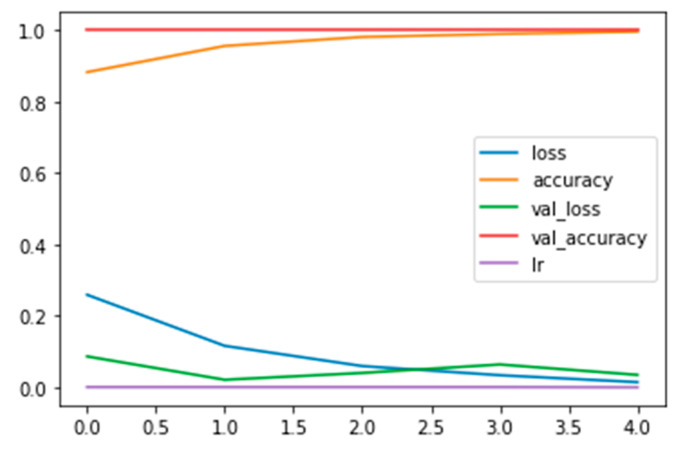
Plot of the training and validation and the loss and accuracy curves, along with the learning rate (see the legend) for the TL_model.

In Figure 5, the plot of the training and validation and the loss and accuracy curves, along with the learning rate (see the legend) for TL_model, is shown.

The accuracy of the TL_model

The testing accuracy is: 91.74603223800659%.

The prediction of the TL_model timestamp

10/10 [==============================]—5 s 219 ms/step

**Figure 6:**
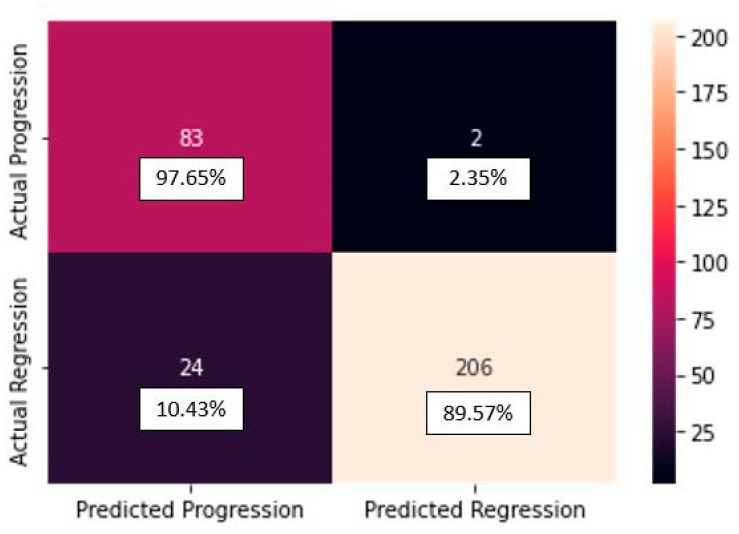
Confusion matrix for the TL_model.

The confusion matrix of the TL_model:

In Figure 6, the confusion matrix for the TL_model from scratch can be seen.

From the confusion matrix for the TL_model, we can see that from the 85 progression cases, the model predicted 83 cases correctly, making only 2 mistakes, predicting regression for cases actually belonging to progression class (false positives). From the 230 regression cases, the model predicted 206 cases correctly, making only 24 mistakes, predicting progression for cases actually belonging to the regression class (false negatives). Also, check the results in the classification report (Table 5).

**Figure 7:**
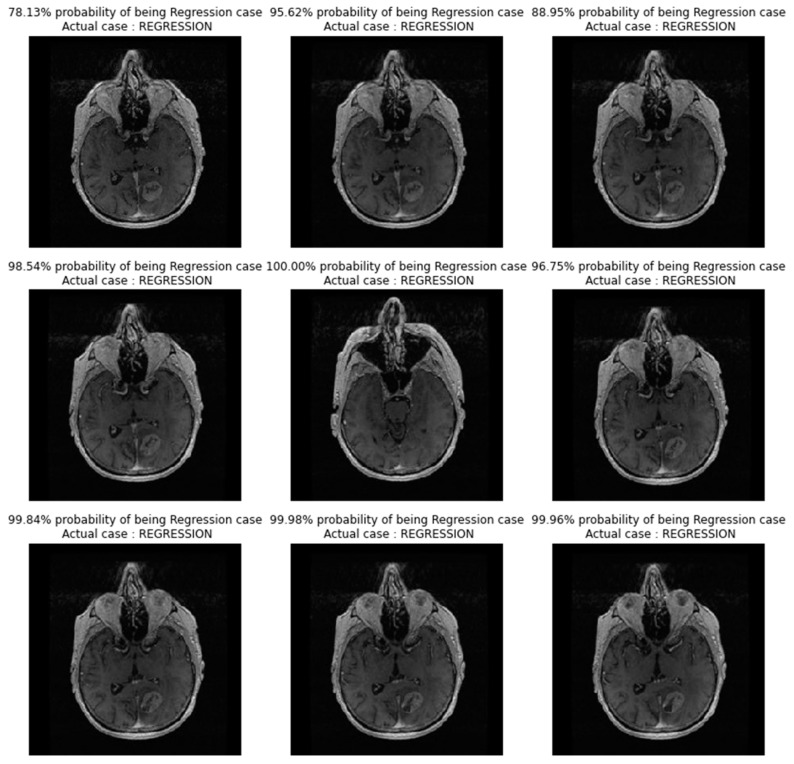
Images showing actual cases versus the predicted cases, with probability of prediction, for the TL_model on unseen images.

In Figure 7, images showing actual cases versus the predicted cases, with the probability of prediction, for the TL_model on unseen images, are presented.

#### 2.4.5. Model-3: Fine Tuning (FT)

The fine tuning technique is the third type of approach for solving this problem. FT is the most efficient and accurate technique because of its flexibility. In this model, every aspect is similar to those in the TL model (Model-2); the only change is the unfreezing of the last few layers of the feature extraction step; all other aspects remain the same. This small change will bring a beneficial results regarding the model prediction because of the retraining the last few layers of the feature learning layers. The pretrained model is also the same as that used in Model-2, which is Resnet152V2 (see Table 6).

*Calculation of parameters for FT Technique:* The Model-3 summary of layers will be the same as for Model-2 because they both employ the same layers up to this point; however, in this technique, the last 15 layers are unfrozen. Due to this change, the number of trainable and non-trainable parameters in this model will also change. It should be noted that the parameters of the dense layers do not change. The total trainable parameters in this technique are 5,789,953, whereas in Model-2, they are 270,593.

##### Fitting and Evaluating the Model (FT)

**Figure 8:**
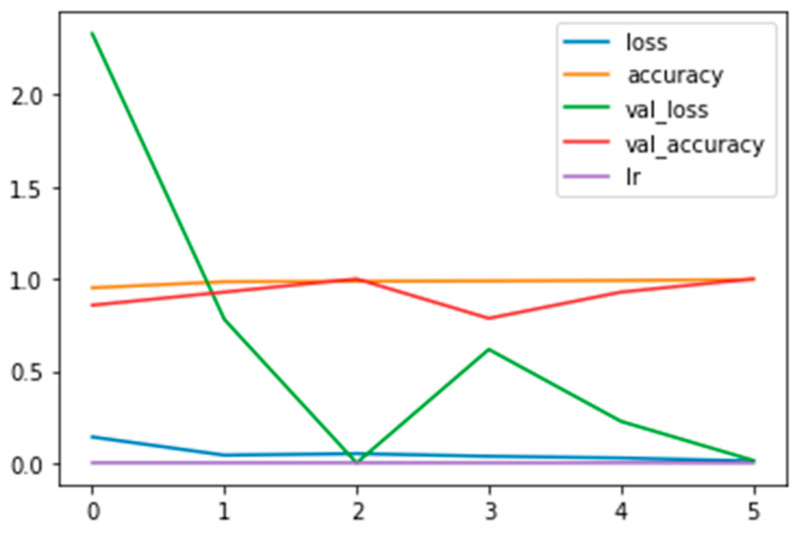
Plot of the training and validation and loss and accuracy curves, along with the learning rate (see the legend) for the FT_model.

In Figure 8, a plot of the training and validation and loss and accuracy curves, along with the learning rate (see the legend) for the FT_model, is shown.

The accuracy of the FT_model:

The testing accuracy is: 83.17460417747498%

The prediction of the FT_model timestamp

10/10 [==============================]—5 s 214 ms/step

**Figure 9:**
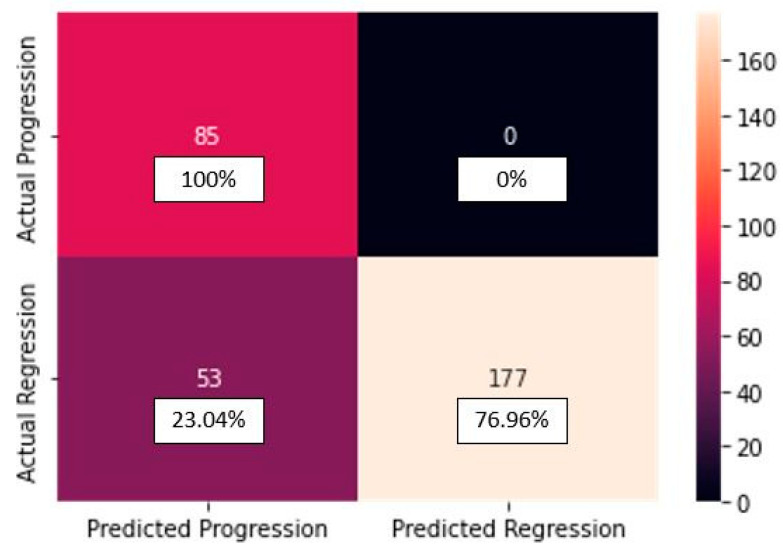
Confusion matrix for the FT_model.

The confusion matrix of the FT_model:

In Figure 9, the confusion matrix for the FT_model from scratch can be seen.

From the confusion matrix for the FT_model, we can see that from the 85 progression cases, the model predicted 85 cases correctly. From the 230 regression cases, the model predicted 177 cases correctly, making 53 mistakes, predicting progression for cases actually belonging to the regression class (false negatives). Please check Table 7 for the classification report.

**Figure 10:**
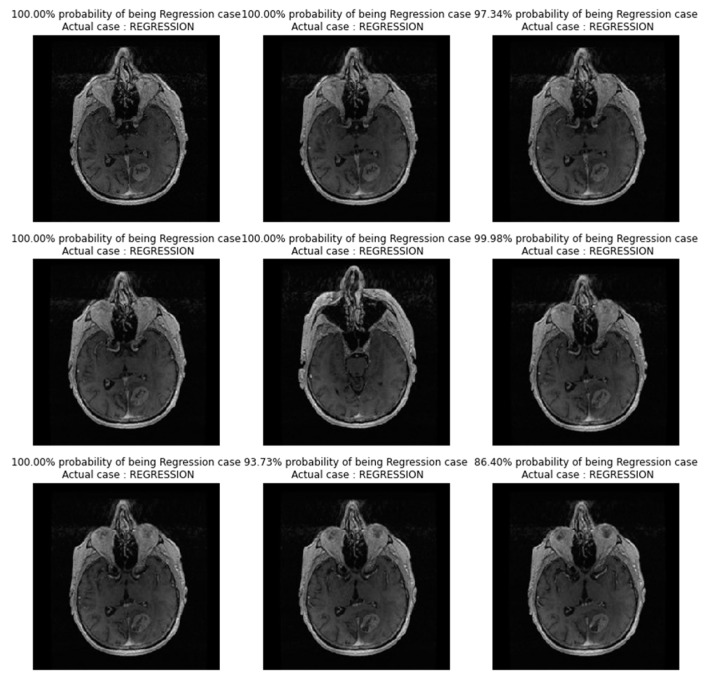
Images showing actual cases versus the predicted cases, with probability of prediction, for the FT_model, on unseen images.

In Figure 10, images showing actual cases versus the predicted cases, with probability of prediction, for the FT_model on unseen images, are presented.

#### 2.4.6. Final Accuracy, ROC Curve, and AUC of Model-1 (CNN Model from Scratch), Model-2 (TL) and Model-3 (FT)

1. The testing accuracy of Model-1 (CNN model from scratch) is: 98.41%.
2. The testing accuracy of Model-2 (transfer learning) is: 91.75%.
3. The testing accuracy of Model-3 (fine tuning) is: 83.17%.

The receiver operating characteristic (ROC) curve is a graphical representation of a binary classifier’s performance as the discrimination threshold varies. It plots the true positive rate (TPR) against the false positive rate (FPR) at different thresholds. 

The area under the ROC curve (AUC) quantifies the binary classifier’s performance over all possible thresholds. It ranges from 0 to 1, with 0.5 indicating random guessing and 1 indicating perfect classification.

To calculate the ROC curve and the AUC, we followed these steps:

Predictions: First, the deep learning model makes predictions for each instance in the dataset. These predictions are often in the form of probability scores for the positive class.

Sorting: Sort the instances based on their predicted scores in descending order.

Threshold Variation: Start with a threshold of 0 (considering all instances as negative) and gradually increase the threshold. At each threshold, calculate the TPR and FPR. 

True Positive Rate (TPR) = True Positives/(True Positives + False Negatives).

False Positive Rate (FPR) = False Positives/(False Positives + True Negatives).

Plotting the ROC Curve: Plot the calculated TPR values on the *y*-axis against the FPR values on the *x*-axis to create the ROC curve.

Calculating the AUC: The AUC is calculated by computing the integral of the ROC curve. This can be done using numerical integration techniques or more simply by summing the areas of the trapezoids formed between adjacent points on the curve.

ROC and AUC are used for:

Robustness to class imbalance: ROC and AUC are less affected by class imbalance than metrics such as accuracy. They provide a comprehensive view of a model’s performance across various classification thresholds.

Threshold Selection: ROC curves help in choosing an appropriate classification threshold based on the desired trade-off between sensitivity and specificity for the specific problem.

Model Comparison: AUC provides an easy way to compare the performance of different models without needing to consider multiple thresholds. A higher AUC generally indicates better discrimination ability.

Insight into Model Behavior: ROC curves reveal how well a model performs at different levels of false positives, which can be important in many real-world applications.

In summary, ROC and AUC are valuable tools for assessing the performance of classification models, especially in situations where class distribution is imbalanced or where different operating points are of interest.

**Figure 11:**
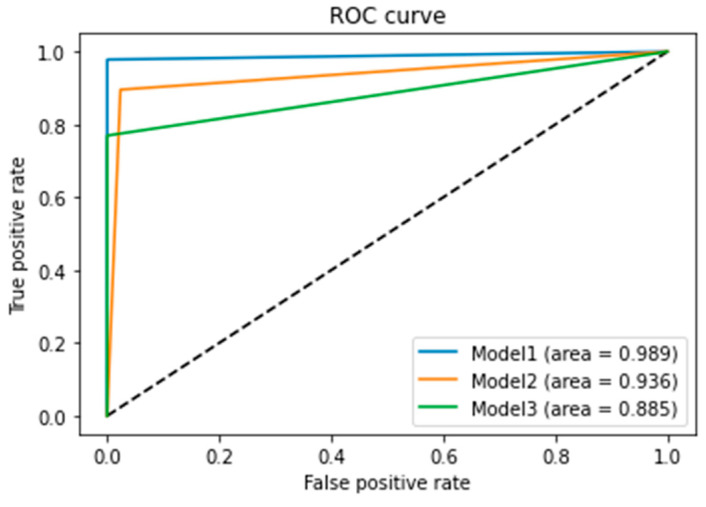
ROC curve and AUC for the three models. AUC of Model-1 (CNN model from scratch), Model-2 (TF), and Model-3 (FT).

Let us analyze the ROC curve and AUC for the three models used (see Figure 11).

The first model, the CNN_model from scratch, achieved an AUC of 0.989, indicating excellent accuracy in distinguishing between positive and negative instances.

The second model, the TL_model, scored 0.936, still performing well, but scoring slightly lower than the first model in distinguishing between positive and negative instances.

The third model, the FT_model, obtained an AUC of 0.885, the lowest among the three models, suggesting that it may not perform as well in distinguishing between positive and negative instances.

Overall, the CNN_model from scratch is the best performer, followed by the TL_model, and then the FT_model. It is important to consider other metrics like precision, recall, and F1-score when evaluating the performance of a binary classification model.

## 3. Discussion and Conclusions

In this paper, we used deep learning techniques to analyze imaging data from patients with stage-treated metastases who underwent Gamma Knife radiosurgery. Our results show that deep learning algorithms accurately predict metastases evolution post-treatment [32].

However, our study has limitations, including a relatively small sample size, a single-center design, and a retrospective nature, potentially introducing biases and confounding factors.

Despite these limitations, our work provides essential insights into using deep learning techniques to predict treatment outcomes in metastases patients, with clinical implications for treatment decision making and patient outcomes. Future studies should validate our models in larger patient cohorts and explore deep learning algorithms in other clinical contexts.

Radiomics, an image analysis technique used in oncology, enhances diagnosis, prognosis, and clinical decision making for precision medicine. In brain metastases, radiomics identifies smaller metastases, defines multiple larger ones, predicts local response post-radiosurgery, and distinguishes radiation injury from metastasis recurrence. Radiomics approaches achieve high diagnostic accuracies of 80–90% [32].

Notable papers related to radiomics and machine learning applications in stereotactic radiosurgery include a comprehensive review discussing brain tumor diagnostics, image segmentation, and distinguishing radiation injury from metastasis relapse [32]. Studies regarding predicting the response after radiosurgery reveal potential, with features like the presence of a necrotic core, the fraction of contrast-enhancing tumor tissue, and the extent of perifocal edema [33,34,35,36,37,38].

The advances in radiomics and deep learning hold promise for precision medicine in brain metastases treatment, enabling precise diagnoses, prognoses, and treatment response monitoring [39].

Randomized trials demonstrate the benefits of SRS as a standalone treatment for brain metastases, without significant decrease in survival. However, SRS alone associates with higher local failure rates, warranting identification of high-risk patients. Radiomics analyses show potential for predicting local failure and SRS response [40,41,42,43,44,45,46,47,48].

Quantitative imaging features correlate with outcomes after radiation therapy, enhancing personalized cancer care. A multidisciplinary approach integrating radiomics and deep learning is essential in the medical decision-making and radiation therapy workflow for bone metastasis [48].

Studies by Huang et al. and others explore significant radiomic features related to core volume and sphericity, predicting local tumor control after GKRS [49]. Machine learning processes predict the brain metastasis response to GKRS, with promising accuracy [50].

Cha et al. developed a radiomics model based on a convolutional neural network to predict the response to SRT for brain metastases, achieving promising results with ensemble models [37].

In conclusion, our deep learning approach accurately predicts metastases evolution. Radiomics and machine learning are promising tools for improving brain metastases treatment. Validation studies and improved integration in clinical workflows are needed to maximize their potential [32,33,34,35,36,37,38,39,40,41,42,43,44,45,46,47,48,49,50,51].

Computational software related to applied fractal analysis used in this study was initiated and then successfully developed in the articles of some of the authors mentioned in the bibliography [51,52,53,54,55].

Classification reports (Table 3, Table 5 and Table 7) for three different deep learning models (CNN_model, TL_model, and FT_model) present performance metrics for a binary classification problem with two classes: “progression” and “regression”.

CNN_model:

- “Progression” class: Precision = 0.94, Recall = 1.00, F1-score = 0.97, Accuracy = 0.98.
- “Regression” class: Precision = 1.00, Recall = 0.98, F1-score = 0.99, Accuracy = 0.98. Overall, the CNN_model performed very well, with high precision, recall, and F1-scores for both classes.

TL_model:

- “Progression” class: Precision = 0.78, Recall = 0.98, F1-score = 0.86, Accuracy = 0.92.
- “Regression” class: Precision = 0.99, Recall = 0.90, F1-score = 0.94, Accuracy = 0.92. The TL_model performed well, with high recall for the “progression” class, but its precision could be improved.

FT_model:

- “Progression” class: Precision = 0.62, Recall = 1.00, F1-score = 0.76, Accuracy = 0.83.
- “Regression” class: Precision = 1.00, Recall = 0.77, F1-score = 0.87, Accuracy = 0.83. The FT_model showed high precision for the “regression” class and high recall for the “progression” class. However, there may be some misclassification in these cases, and the F1-scores indicate a tradeoff between precision and recall.

In summary, all three models demonstrated good to very good performance, but there is room for improvement in certain aspects. Further analysis, such as examining the confusion matrix, may provide additional insights into the models’ strengths and weaknesses.

## Figures and Tables

**Table 1:** The splitting of the BrainMet image dataset in TRAIN, TEST, VAL and the number of images belonging to the two classes.

	TRAIN Set	TEST Set	VALIDATION Set
	2865	315	14
Progression	782	85	7
Regression	2083	230	7

**Table 2:** Parameters in the first model, CNN model from scratch. Model: “sequential”.

Layer (Type)	Output Shape	Param #
conv2d (Conv2D)	(None, 222, 222, 16)	448
max_pooling2d (MaxPooling2D)	(None, 111, 111, 16)	0
conv2d_1 (Conv2D)	(None, 109, 109, 32)	4640
max_pooling2d_1 (MaxPooling2	(None, 54, 54, 32)	0
conv2d_2 (Conv2D)	(None, 52, 52, 32)	9248
max_pooling2d_2 (MaxPooling2	(None, 26, 26, 32)	0
conv2d_3 (Conv2D)	(None, 24, 24, 64)	18,496
max_pooling2d_3 (MaxPooling2	(None, 12, 12, 64)	0
conv2d_4 (Conv2D)	(None, 10, 10, 64)	36,928
max_pooling2d_4 (MaxPooling2	(None, 5, 5, 64)	0
flatten (Flatten)	(None, 1600)	0
dense (Dense)	(None, 128)	204,928
dense_1 (Dense)	(None, 64)	8256
dense_2 (Dense)	(None, 1)	65

Total params: 283,009; trainable params: 283,009; non-trainable params: 0.

**Table 3:** Classification report for the CNN_model from scratch.

	Precision	Recall	F1-Score	Support
Progression	0.94	1.00	0.97	85
Regression	1.00	0.98	0.99	230
Accuracy	-	-	0.98	315
Macro avg	0.97	0.99	0.98	315
Weighted avg	0.99	0.98	0.98	315

**Table 4:** Parameters of the second model, TL model.

Layer (Type)	Output Shape	Param #
input_1 (InputLayer)	[(None, 224, 224, 3)]	0
resnet152v2 (Functional)	(None, 7, 7, 2048)	58,331,648
global_average_pooling2d (Gl)	(None, 2048)	0
dense (Dense)	(None, 128)	262,272
dense_1 (Dense)	(None, 64)	8256
dense_2 (Dense)	(None, 1)	65

Total params: 58,602,241; trainable params: 270,593; non-trainable params: 58,331,648.

**Table 5:** Classification report for the TL_model.

	Precision	Recall	F1-Score	Support
Progression	0.78	0.98	0.86	85
Regression	0.99	0.90	0.94	230
Accuracy	-	-	0.92	315
Macro avg	0.88	0.94	0.90	315
Weighted avg	0.93	0.92	0.92	315

**Table 6:** Parameters of the third model, FT model.

Layer (Type)	Output Shape	Param #
input_1 (InputLayer)	[(None, 224, 224, 3)]	0
resnet152v2 (Functional)	(None, 7, 7, 2048)	58,331,648
global_average_pooling2d (Gl)	(None, 2048)	0
dense (Dense)	(None, 128)	262,272
dense_1 (Dense)	(None, 64)	8256
dense_2 (Dense)	(None, 1)	65

Total params: 58,602,241. Trainable params: 5,789,953. Non-trainable params: 52,812,288.

**Table 7:** Classification report for the FT_model.

	Precision	Recall	F1-Score	Support
Progression	0.62	1.00	0.76	85
Regression	1.00	0.77	0.87	230
Accuracy	-	-	0.83	315
Macro avg	0.81	0.88	0.82	315
Weighted avg	0.90	0.83	0.84	315

## Data Availability

Data is unavailable due to patient privacy and ethical restrictions.
